# Annual Report of the Commissioners, Superintendent, and Treasurer of the Indiana Hospital for the Insane; for the Year 1863

**Published:** 1864-04

**Authors:** 


ANNUAL REPORT OF THE COMMISSIONERS, SUPERINTENDENT, AND TREASURER OF THE INDIANA HOSPITAL FOR THE INSANE; FOR THE YEAR 31ST, 1663.

